# Understanding the self of people with dementia: Psychological and philosophical considerations

**DOI:** 10.1007/s00391-020-01718-1

**Published:** 2020-03-23

**Authors:** Fabian Hutmacher

**Affiliations:** Lehrstuhl für Psychologie VI, Universität Regensburg. Correspondence concerning this article should be addressed to Fabian Hutmacher, Lehrstuhl für Psychologie VI, Universität Regensburg, Universitätsstraße 31, 93053 Regensburg, Germany

**Keywords:** Dementia, Life story, Remembering self, Experiencing self, Body memory, Demenz, Lebensgeschichte, Erinnerndes Selbst, Erlebendes Selbst, Leibgedächtnis

## Abstract

**Background:**

The ability to create and maintain an ongoing life narrative is a key characteristic of what makes us human; however, people with dementia lose this ability in the course of the disease. If the notion of having a self is intimately linked with being able to create and maintain a life narrative and if people with dementia tend to lose this ability, what kind of self do people with dementia still possess?

**Objective and methods:**

Recent theories developed in psychology and philosophy suggest that at least two notions of the self have to be distinguished: the experiencing self and the remembering self. This distinction is applied to people with dementia.

**Results:**

While the remembering self is impaired in people with dementia, their experiencing self remains relatively intact. Critically, the experiencing self is a self with inner depth, mirroring the life history of the individual. Thus, the self of people with dementia is not unextended in time. Although people with dementia may have lost the ability to tell a story about their life, they are still able to express this story in their behavior.

**Conclusion:**

Understanding the structure of the self of people with dementia can help guide interactions as well as the designing of appropriate interventions and environments. Moreover, realizing the inner depth of the self of people with dementia may help acknowledge their dignity and personhood.

## Abstract (de)

**Hintergrund:**

Die Fähigkeit, unser Leben als Geschichte zu erzählen, ist ein entscheidendes Merkmal dessen, was uns als Menschen ausmacht. Menschen mit Demenz verlieren diese Fähigkeit jedoch im Laufe der Krankheit. Wenn die Vorstellung, ein Selbst zu haben, eng mit der Fähigkeit verbunden ist, unser Leben als Geschichte zu erzählen und wenn Menschen mit Demenz dazu neigen, diese Fähigkeit zu verlieren, welche Art von Selbst besitzen Menschen mit Demenz dann noch?

**Ziele und Methoden:**

Aktuelle Theorien aus Psychologie und Philosophie legen nahe, dass mindestens zwei Vorstellungen des Selbst unterschieden werden müssen: das „erlebende Selbst“ und das „erinnernde Selbst“. Diese Unterscheidung wird auf Menschen mit Demenz angewendet.

**Ergebnisse:**

Während das erinnernde Selbst bei Menschen mit Demenz beeinträchtigt ist, bleibt das erlebende Selbst vergleichsweise unbeeinträchtigt. Entscheidend dabei ist, dass es sich beim erlebenden Selbst um ein Selbst mit innerer Tiefe handelt, das die Lebensgeschichte des Individuums widerspiegelt. Das Selbst von Menschen mit Demenz ist also nicht zeitlich unausgedehnt. Obwohl Menschen mit Demenz möglicherweise nicht mehr in der Lage sind, eine Geschichte über ihr Leben zu erzählen, können sie diese Geschichte immer noch in ihrem Verhalten ausdrücken.

**Schlussfolgerung:**

Die Struktur des Selbst bei Menschen mit Demenz zu verstehen, kann dabei helfen, Interaktionen zu lenken sowie geeignete Interventionen und Lebensumwelten zu entwerfen. Darüber hinaus kann ein Verständnis für die innere Tiefe des Selbst von Menschen mit Demenz dazu beitragen, ihre Würde und ihr Personsein anzuerkennen.

## Brief introduction

We do not like to think of our life as an accumulation of random, unrelated events. Rather, we want our life to be a meaningfully evolving story that we can tell others, as well as ourselves; however, the ability to tell stories about one’s own life and to perceive one’s own life as a coherent story is dramatically decreased in people with dementia, especially in the later stages of the disease. If the notion of having a self is intimately linked with being able to create and maintain a life narrative, and if people with dementia tend to lose this ability, what kind of self do people with dementia still possess?

## Introduction

Despite the significant differences between the various forms of dementia, in all of them the deterioration of memory and language is a key characteristic. This seems to endanger the idea that humans are storytellers and that the stories we tell define who we are [21]. Thus, at first sight, one may argue that the self of people with dementia is a severely fragmented and reduced self, which becomes more fragmented and reduced the more the disease progresses. Such an understanding of the self of people with dementia is in line with contemporary discourses on dementia, which often metaphorically depict dementia as a “return to childhood” or as “living death” [26]. While the first metaphor expresses a more positive evaluation of the disease than the latter, both share the assumption that people with dementia have a severely impaired self, either because they relive the seemingly innocent state of childhood or because they are practically dead already, zombies without inner depth. On closer examination though, things turn out to be more complicated and complex. As the first section demonstrates, there are different notions of self, among which the narrative, remembering self is only one. Thus, although the narrative self may be impaired in people with dementia, this does not mean that the self of people with dementia is a blank slate, a self without history or depth. Based on this idea, the second section describes and discusses the depths of the self of people with dementia, while the third and last section tentatively explores some conclusions that can be drawn from these insights for engaging with people with dementia.

## Different notions of self

Imagine listening to the recording of a symphony orchestra. Unfortunately, the disc has a scratch near the end, producing a shocking sound. Hearing that sound does not only make you cringe, it also overshadows the minutes before. When someone asks you whether you liked the recording, you may no longer think about the wonderful piece of music that you were listening to but rather about the horrible sound that disturbed you near the end. It seems that the sound ruined the whole experience; however, according to Kahneman [16], who uses this example to distinguish between two kinds of selves, the *experiencing self* and the *remembering self*, the scratch does *not* in fact ruin the whole experience. It ruins the *memory of the experience*, it ruins what the remembering self thinks about the experience when looking back on it, but not what the experiencing self perceived while listening to the music, i.e., while *making the experience* up until the shocking sound.

What Kahneman calls the experiencing self, has also been described as the *core* or *minimal self*, and what he defines as the remembering self, has also been characterized as the *extended* or *narrative self* (for an overview see, e.g., [11, 30]; for the historical origins see the distinction between “I” and “Me” by William James [14]). While the narrative, remembering self can be defined as the “more or less coherent self […] that is constituted with a past and a future in the various stories that we and others tell about ourselves” [11, p. 231], the minimal, experiencing self is defined as the self which makes experiences in the present moment. What can we learn from this distinction in the case of people with dementia?

Although neither Kahneman nor the proponents of the distinction between a minimal and a narrative self explicitly refer to dementia in their writings, one could argue that dementia primarily affects the remembering self, i.e., the ability to put different experiences in context and to perceive different events as belonging to the same story, while the experiencing self remains largely unaffected. This idea fits well with the differentiation between *vivre* and *se vivre* introduced by Tatossian [29] to describe the state of mind of people with dementia. Following his line of thought, people with dementia are still aware of what is happening right now (vivre), but increasingly have problems with taking a distanced and reflective stance towards these momentary experiences (se vivre); however, this distinction should not be taken to the extreme for at least two reasons. First, people with dementia still show episodes of lucidity from time to time (see, e.g., [23]) or as Post [24] put it: “The reality is that until the very advanced and even terminal stage of dementia, the person with dementia will usually have sporadically articulated memories of deeply meaningful events and relationships ensconced in long-term memory” (p. 231). Secondly, people with dementia often seem to feel the need to locate themselves within time [22], indicating an urge to create stability and orientation reaching beyond the present moment [28]. As these two arguments demonstrate, claiming that the remembering self disappears completely in people with dementia would oversimplify the matter [15].

Nonetheless, these caveats do not touch upon the general idea that it is primarily the remembering self that is affected by dementia while the experiencing self remains largely intact; however, if the minimal, experiencing self plays such a crucial role in people with dementia, it seems advisable to take a closer look at the concept: how can it be characterized in more detail? Gallagher [11] for his part seems to believe, for instance, that there is not much detail to be added. For him, the minimal self is “the consciousness of oneself as an immediate subject of experience, unextended in time” (p. 15). The idea that the minimal, experiencing self is unextended in time, that it consists of nothing but the *mineness*, “that is, the fact that the experiences are characterized by a first-personal givenness that immediately reveals them as one’s own” [30, p. 124], would imply that the self of a person with dementia is a self with no inner depth, were it not for the few fragments of the remembering self that still remain. In contrast, some authors have argued that the minimal, experiencing self is spatially-temporally structured and influenced by the history of the individual (see, e.g., [10, 27, 30]). But what does that mean?

## The depths of the experiencing self

We do not only have semantic-conceptual memories, i.e. verbalizable memories about facts and ideas as well as our own lives, but also perceptual memories, i.e. memories of perceptual information that has reached our senses. Take the abovementioned example of listening to the recording of a symphony orchestra: You may know that you will like this specific recording because you are a fan of classical music or because the orchestra was conducted by your favorite conductor. In this case, you are using conceptual knowledge about your musical preferences. In a different situation, for example when driving on the highway and trying to select a radio station, you do not have to think about the different kinds of music that you like (and those that you dislike) in order to make a choice. You will just stop switching between different radio stations once you hear a piece of music that sounds familiar or pleasant to you. When referring to perceptual memories, Fuchs [9, 10] used the term *body memory*. According to him, there are six different forms of body memory, all of which he believes to be present in people with dementia, even in the later stages of the disease [10]. If this were true, the experiences of the minimal self and its reactions to these experiences would be guided by the prior life history of the individual. Thus, the minimal, experiencing self in people with dementia would indeed be a self with inner depth. The conclusions that can be drawn from this idea are discussed in the final section. First, let me describe the different forms of body memory and the way they are supposedly still present in people with dementia.

*Procedural memory* is memory for “patterned sequences of movement, well-practiced habits, skillful handling of instruments, as well as familiarity with patterns of perception” [9, p. 12]. People with dementia are able to handle everyday tools (e.g., a toothbrush) and to perform everyday actions (e.g., brushing their teeth) even when they have already forgotten the names of the tools. In fact, they may still be able to learn new procedures like dance moves [5, 25]. *Situational memory* is—as the name already suggests—memory for specific, well-known situations and places. A person now suffering from dementia who has been a football fan for his or her whole life, for instance, may still be able to enjoy the atmosphere in a football stadium and to cheer for the home team, although he or she may no longer be capable of recalling the name of the players or the football club. In a similar manner, people with dementia may still lower their voices when entering a church without being able to explain why this is considered to be the appropriate behavior or to navigate through their own home without being able to draw or to describe it properly (see e.g., [19]). The third form of body memory, *intercorporeal memory*, refers to the fact that our ways of interacting with people are shaped by our prior experiences in social situations. We know how to behave when someone is sad or happy, and we are able to sense whether someone wants to be left alone or would prefer a hug. In fact, people with dementia seem to be very sensitive to subtle changes in the atmosphere of the situation as well as the emotional state of the person they are interacting with and still show their own feelings and needs in their gestures and facial expressions long after they have lost the ability to express them verbally [10]. The fourth form of memory, *incorporative memory*, strongly resembles what Bourdieu [2] called the *habitus*. The habitus “denotes the entire social appearance of a person including his or her posture, manners, taste, clothing, attitudes and general way of life” [9, p. 16]. In this sense, people with dementia preserve their typical behavior when expressing emotions, showing interest or disgust, joy or shame. The fifth and sixth form of body memory, *pain memory* and *traumatic memory* are somewhat related: Unpleasant memories of the past largely influence our present thoughts and actions, even when we cannot verbalize them (see [3] for an early case study). This applies to both mildly painful and extremely painful, traumatic memories. A person with dementia, who had to hide in the basement of the house during one of the bombing raids of World War II, for instance, may refuse to go to the basement of the nursing home without being able to give their reasons for this behavior.

## The alterity of the self of people with dementia

The self of people with dementia is not unextended in time: Although the remembering self may be gradually fading away, the remaining experiencing self is a self with inner depth, mirroring the life history of the individual. That is not meant to dispute the significant burdens, challenges, and difficulties that come with a dementia diagnosis for both the diseased person as well as family members and friends. Nevertheless, understanding the complexity of the self of people with dementia can help to counterbalance our bias towards the rational and intellectual understanding of who we are. Humans are not only storytellers; humans are also beings with an extremely rich and embodied inner perceptual world (for recent empirical studies with healthy subjects, see, e.g., [12, 13]). This insight has a number of important implications for engaging with people with dementia. First, it would be shortsighted to describe people with dementia as being endangered of losing their self or even of “‘unbecoming’ a self” [8, p. 36]. The remembering self of people with dementia does indeed become increasingly fragmented during the course of the disease while the experiencing self does not or at least to a far smaller degree. Keeping this in mind seems to be of greatest importance as it has been shown that treating people with dementia as if they had already lost their sense of self, may work as a self-fulfilling prophecy, i.e. it may accelerate the disease [1, 17]. Second, losing the abilities of the remembering self, i.e. losing the ability to narrate one’s own life, probably scares many people. It does so because we tend to think of ourselves as storytellers. Acknowledging the importance of body memory can help us to correct this bias and to perceive the perspective of people with dementia as a perspective *in it*’*s own right*. This has important ethical consequences: when living with dementia is characterized as a radically different state of mind, centered around the experiencing rather than the remembering self, and not so much or at least not solely as a deficient, deteriorated condition, there is reason to believe that terms like autonomy and personhood are still meaningful and that the possibility of encountering other people remains of vital importance, even for people with later stage dementia. Third, our notion of well-being is dominated by the perspective of the remembering self (see [16]): Whether we are happy with our life largely depends on the way we evaluate our life as a whole. For people with dementia, however, the experiential dimension of well-being may become more important. Thus, although the loss of the remembering self may be experienced as negative by people with dementia, living with dementia does not necessarily mean leading an unhappy life. Although symptoms of depression and anxiety are admittedly quite common among people with dementia, it can be shown that even “those who are, from a cognitive standpoint, severely demented” [18, p. 269] can and do experience well-being. Fourth, the shift from the remembering to the experiencing self needs to result in different kinds of interactions with people with dementia. In fact, recognizing and accepting the person’s reality instead of trying to correct it is one of the core principles in person-centered dementia care (for recent reviews see, e.g., [6, 20]). Forcing a narrative upon people with dementia, which they cannot relate to, either because they have forgotten that this narrative used to be the narrative of their life or because this narrative has simply lost its importance, is frustrating for all people involved. Instead, it seems important to validate the momentary feelings, needs, and perceptions of the individual. An interesting concept in this respect is the so-called companionship of themes of being (see, e.g., [4]). Fifth, the knowledge about the structure of the self of people with dementia can inform the development of adequate interventions and environments. The architecture of nursing homes, for instance, should be and is in fact adjusted to the needs and perceptual habits of people with dementia [7]. Besides general rules of thumb regarding the design of well-suited environments, it seems important to acquire profound knowledge about the *individual* person with dementia. Although people with dementia may benefit from sensory stimulation and from a rich perceptual environment in general, they will probably benefit even more when the stimulation is aligned with their prior sensory experiences and present preferences. Thus, knowing the person with dementia, i.e., knowing past and present beliefs and needs, likes and dislikes, is another aspect in person-centered dementia care for a good reason.

In short, dementia is neither a return to childhood nor a living death. Although the disease disrupts well-established ways of interacting with each other and thus profoundly changes the relationship between the diseased person and his or her family and friends, the self of people with dementia is not a blank slate. Rather, people with dementia still have access to a complex life history.

## Practical conclusion

- Impairments of the remembering self do not imply that people with dementia possess only a minimal self with no inner depth. Rather, their experiencing self mirrors the story of their lives, which they may no longer be able to express verbally.
- Personhood is not bound to the ability to explicitly recall one’s past in a coherent manner. Although the self of people with dementia changes profoundly, they are not unbecoming a self—an idea that is well in line with the principles of person-centered dementia care.
- People with dementia may benefit from sensory stimulation and from a rich perceptual environment, and especially from an environment that is aligned with the sensory history of their lives.
- When trying to get in touch with people with dementia, it may be advisable, especially in the later stages of the disease, to do so by focusing on bodily interactions, present needs and shared perceptions.
